# Circulating markers of bone turnover

**DOI:** 10.1007/s40620-017-0408-8

**Published:** 2017-05-13

**Authors:** Marc G. Vervloet, Vincent M. Brandenburg, on behalf of CKD-MBD working group of ERA-EDTA, Jordi Bover, Vincent Brandenburg, Adrian Covic, Mario Cozzolino, Pieter Evenepoel, David Goldsmith, Ziad Massy, Sandro Mazzaferro, Pablo Ūrena-Torres, Marc Vervloet

**Affiliations:** 1 Department of Nephrology and Institute of Cardiovascular Research, VU University Medical Center, De Boelelaan 1117, 1081 HV Amsterdam, The Netherlands; 2 Department of Cardiology, RWTH University Hospital Aachen, Aachen, Germany

**Keywords:** Biomarkers, Bone turnover, Chronic kidney disease, CKD-MBD

## Abstract

Renal osteodystrophy is a feature of chronic kidney disease (CKD), with increasing prevalence as CKD progresses. This bone disease is responsible for major morbidity, including fractures, and a deterioration in the quality of life and its sequelae. Circulating biomarkers of renal osteodystrophy typically indicate bone turnover, but not other features of bone, like bone volume, mineralization, quality or strength. Bone turnover can be considered to be primarily a reflection of bone cell activity, in particular that of osteoblasts and osteoclasts. Since current treatments for bone disease usually target cellular activity, biomarkers are considered to be able to contribute to the decision-making for treatment and its follow-up. In CKD, one has to consider the impact of a diminished clearance of biomarkers or their altered metabolism, both potentially limiting its clinical use. Here, several aspects of the most frequently used biomarkers of bone turnover are reviewed, with an emphasis on the specific situation represented by CKD. This review is based on the overview lecture at the symposium held in Amsterdam, September 23, 2016: “The Bone In CKD”, organized by the CKD-MBD working group of ERA-EDTA.

## Introduction

For decades it has been acknowledged that chronic kidney disease (CKD) is associated with bone disease and, after a causal link was established, this bone disease was termed renal osteodystrophy (ROD) [1]. As outlined in detail elsewhere in this issue, ROD encompasses a wide spectrum of bone histological abnormalities, recently categorized based on three parameters: rate of turnover, amount of mineralization and bone volume (TMV) [2]. Although novel imaging techniques are promising in terms of their ability to noninvasively quantify components of ROD—like dual-energy X-ray absorptiometry (DEXA) for combined bone volume and mineralization and quantitative computerized tomography (CT) for bone volume and architecture—they are usually not capable of measuring bone turnover. Bone turnover generally indicates activity of bone cells, since turnover by definition is a dynamic biological process, which contrasts with mineralization which is a more passive physicochemical process. Since dynamic processes are difficult to estimate on a single time point, this feature of bone biology can be assessed by bone histomorphometry using tetracycline double-labeling, where both the width between the two labels and the length of each label is a proxy for time, and therefore for the osteoid volume formation rate over a given time [3, 4]. Currently, many available treatment options for ROD, such as calcimimetics, bisphosphonates, denosumab, and teriparatide, target bone turnover, with bone volume and strength as a net resultant of the intervention on osteoclasts and osteoblasts. For these reasons, longitudinal assessment of bone turnover is of relevance in clinical decision-making to select and initiate treatment and to monitor its effect. Although bone histomorphometry is considered the gold standard, it has important limitations with regard to meeting this clinical need. Among these limitations are the potential of sampling error since bone formation is a cyclic process which includes a period of quiescence [4], invasiveness, costs, limited availability of both the technique and assessment of the samples, and barriers to perform serial biopsies in individual patients.

**Fig. 1:**
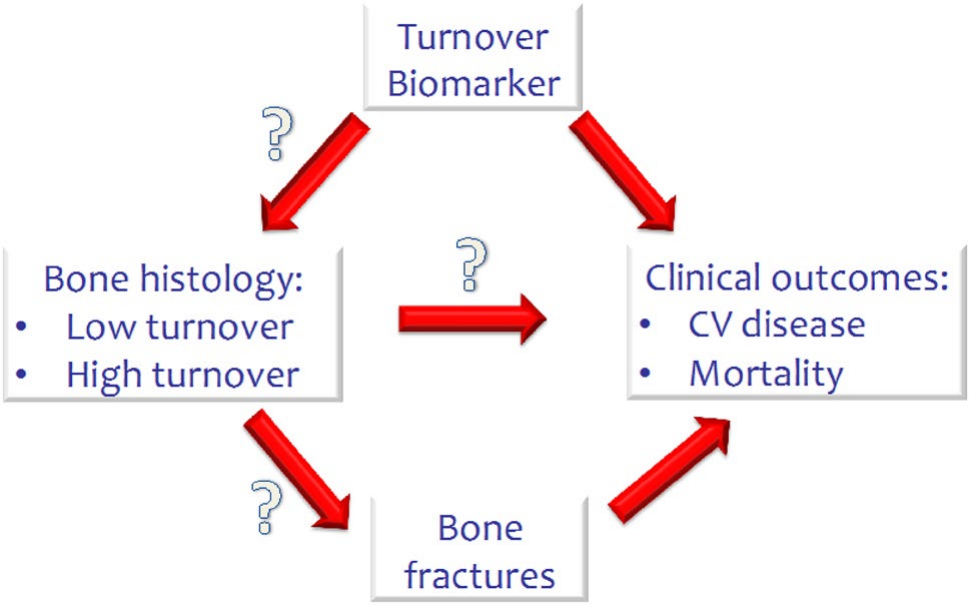
Complex relationship between biomarkers of bone turnover and clinical outcome. For several biomarkers, like PTH and alkaline phosphatase, the association with mortality and cardiovascular (CV) morbidity is reasonably well established. However, the association between these markers and bone histomorphometry is less clear. Importantly, no strong data clarify the relationship between bone histomorphometry and either future bone fracture or CV complications, mostly due to a paucity of data examining bone histology

For these reasons, circulating markers, that can be assessed in the blood compartment, are attractive alternatives to some aspects of bone histomorphometry, in particular turnover. Indeed, several biomarkers of renal osteodystrophy are being used clinically or in clinical studies, as will be addressed below. Besides the attractiveness of these biomarkers due to their ease of measurement, it is also important to keep several limitations in mind that are applicable to all these humoral factors. As for all biochemical variables, the clinical relevance depends on either the marker’s predictive power for incident clinical events (like future fractures or cardiovascular events) or its causal role in the pathogenesis of ROD, because only in the latter case does the process as reflected by the biomarker qualify as a treatment target. For none of the currently available bone markers is this established beyond doubt. In addition, biomarkers are used clinically both as a proxy to establish the type of bone disease or quantify its severity, and also to assess (cardiovascular) risk, as summarized in Fig. 1. Interestingly, some so-called bone-turnover markers may not be specific for indicating metabolic processes in bone tissue only, like sclerostin or bone-specific alkaline phosphatase (BSAP), but can also be expressed in cells of cardiovascular tissues [5–7], and finally these markers may in some pathological states be a marker for non-primary bone disease, like total alkaline phosphatase (AP) in liver disease and parathyroid hormone (PTH) in primary hyperparathyroidism. Another important issue when considering measuring a humoral factor as an indicator of bone turnover is that the kinetics of these markers in serum differs substantially from the place where bone formation occurs [4, 8]. In addition, an inherent complication of CKD is that interpretation of substance concentrations is complicated by the reduced renal clearance of that substance, which thereby no longer reflects its production rate. Finally, as mentioned above, most circulating biomarkers are a reflection only of bone turnover, and not of more static features like mineral density, bone quality and strength.

## The interplay between bone markers

**Fig. 2:**
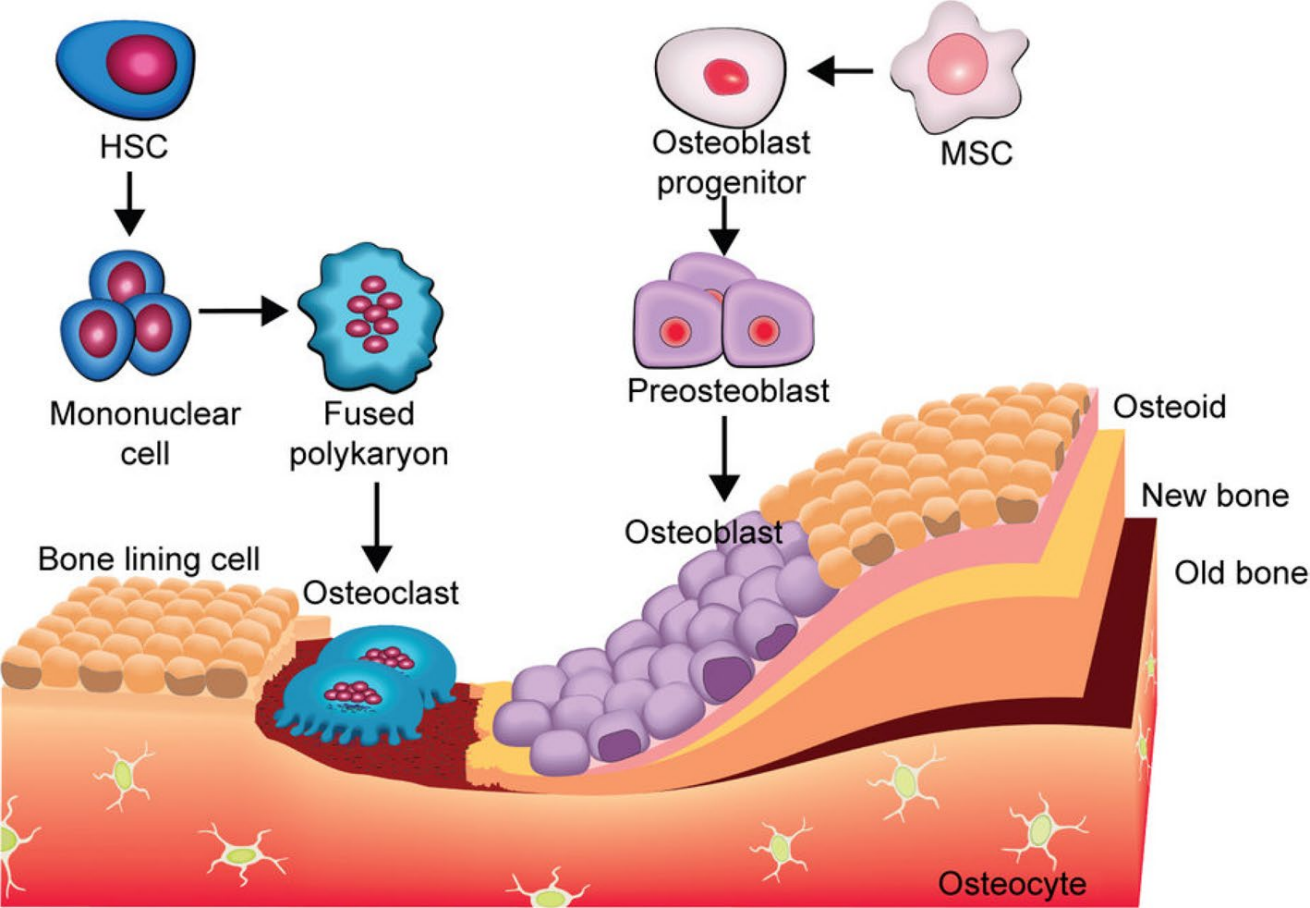
Despite the different origin of osteoblasts on the *right* and osteoclasts on the *left*, their activity is highly coordinated and under cellular control orchestrated by osteocytes, hidden in mineralized bone and a highly complex system of paracrine action humoral factors, not shown in the diagram. (From [22]: approval pending)

Several bone markers differ as to their origin and vary substantially by nature. Some are direct regulators of bone formation, like PTH (produced outside bone) and sclerostin, while others are proteins produced by bone cells as locally acting factors, spilled into systemic circulation, like BSAP and tartrate-resistant acid phosphatase 5b (TRAP5b), or by-products of either production or cleavage of bone collagen, like the N-terminal domain of the propeptide of procollagen 1 (P1NP) and C-terminal crosslaps (CTX), respectively. Due to these different backgrounds, it can be expected that there is no clear correlation between biomarkers even if they reflect the same biological process in bone. Indeed, while PTH can be considered a driver of bone turnover, its association with BSAP, an indicator of osteoblastic activity, is generally vague in patients on dialysis when longitudinally assessed [9]. This may in part be explained by CKD-associated PTH-resistance, which can consist of either posttranslational modification of PTH rendering it biologically inactive, or end-organ resistance to its actions [10]. Alternatively, the differences in kinetics of two separate indicators of bone formation, as outlined previously, may also explain in part why bone markers indicating the same feature of bone biology may dissociate. Generally, when estimating bone turnover, this is done by a biomarker that indicates either formation or resorption of bone. The underlying assumption that these are in balance is usually true [4, 11] as activity of osteoblasts and osteoblast is in balance in most circumstances (Fig. 2). In that case, measuring bone formation also reliably estimates the bone resorption rate. In pathological states, however, this may be different, as in postmenopausal women [12, 13] or during use of glucocorticosteroids [14, 15]. Very relevant for CKD in this regard is the fact that in treated and untreated secondary hyperparathyroidism the bone formation rate may also be dissociated from bone resorption [16]. It is well established that in severe hyperparathyroidism, bone balance is usually negative despite the high level of the hormone that promotes osteoblast activity, and in turn bone mass increases after correction of overt hyperparathyroidism [17]. For these reasons, especially in CKD, it is important to realize that estimating bone turnover by measuring circulating biomarkers is full of pitfalls, that even a reliable estimate of turnover does not indicate changes in bone balance, and that fracture risk is also dependent on bone features that cannot be assessed by biomarkers, nor even by bone histomorphometry like architecture and bone strength. With these considerations in mind, in the remainder of this article we will address a selected number of circulating markers of bone turnover, that are most used or promising. Some of the markers listed below may contribute meaningfully to every-day clinical practice in ROD assessment and therapy. There are several additional biomarkers available, such as osteocalcin, pyridinolines, sclerostin and c-terminal propeptide of procollagen 1 (P1CP), but the authors currently attribute the strongest clinical relevance and evidence to PTH, AP (BSAP), P1NP, CTX, and TRAP5b when assessing bone turnover. Moreover, the pyridinolines (and the related deoxypyridinoline) are usually measured in the urine and therefore heavily rely on kidney function, and as such are unreliable in that setting [18]. Osteocalcin, although bone derived, and as such also used as a bone turnover marker, exists in various carboxylation states, which require dedicated assays to distinguish. Importantly, this carboxylation status depends on vitamin K status, which is variable in CKD, thereby losing its value as a bone turnover marker [19]. Several forms appear better indicators for different processes, like atherosclerosis [20]. Sclerostin, though definitely involved in bone formation, is far from being a valuable additional biomarker of bone turnover [21].

## Parathyroid hormone

Undoubtedly PTH is the most frequently used biomarker to estimate bone turnover. The clinical application of PTH as a biomarker is attractive because it is readily available, routinely used and, importantly, modifiable. This latter virtue, by either vitamin D, phosphate homeostasis or calcimimetics, holds the prospect of modulating bone turnover itself. Very different from most other turnover markers, PTH is not produced in bone tissue and its secretion is not dictated by local demand in bone as sensed by mechanical forces by osteocytes in bone, as is the case for several other indicators of bone turnover [23, 24]. Guidelines in nephrology provide PTH target ranges [25]. The definition of these ranges, however, is predominantly based on association studies between PTH and mortality, as such reinforced by recent observations [26], but not by bone turnover or fracture risk. Indeed using the Kidney Disease Improving Global Outcomes (KDIGO) target range for CKD stage 5D (2–9 times the upper normal limit for the assay used) is clinically useless to identify either low or high bone turnover disease (see Table 1) [27]. To estimate the validity of PTH as an indicator of bone turnover, the gold standard should be bone histomorphometry ideally with future fracture risk. Indeed, several previous studies have examined this issue. Generally, there was a significant trade-off between positive predictive value (PPV) to detect either low or high turnover bone disease, and sensitivity. Exemplary for this dilemma is the study by Torres et al. [28]. In 119 patients with advanced CKD, bone turnover was assessed by bone histomorphometry. An important strength of this study was that patients were unselected by indication to undergo bone biopsy. With a PTH value above 450 pmol/l the PPV was 100% to diagnose high turnover disease, but the sensitivity was only 43%, indicating that applying this PTH value, more than half of high bone turnover cases were missed. Alternatively, using a lower threshold for PTH the misclassification of high turnover disease increases, but there are less missed cases [29]. This same trade-off exists for defining low-turnover disease based on PTH values [25]. The recent well-performed Bonafide study evaluated bone biopsy in patients selected by concentrations of PTH, BSAP and calcium (above 300 pg/dl, 20.9 ng/dl and 8.4 mg/dl, respectively) and followed these subjects after starting treatment with a calcimimetic [16]. The PPV at baseline for this constellation of biomarkers was 110 confirmed cases out of 135 evaluable biopsy samples, yielding a PPV of 81%, but by design the sensitivity could not be determined. Among the largest studies to evaluate diagnostic accuracy of PTH in dialysis patients is a very recent pooled analysis of data from four countries [27]. PTH, among other biomarkers, was centrally assessed from stored serum samples using a second and third generation assay in parallel. The optimal level for intact PTH (iPTH) to discriminate low from non-low turnover disease was 104 pg/mg and high versus non-high had an optimal concentration of 323 pg/ml (upper limit of normal for the applied Roche assay: 65 pg/ml). Although the area under the receiver operating curve for iPTH was only 0.701 and 0.724, indicating borderline clinical usefulness, iPTH was, however, not outperformed by other biomarkers, including the third generation PTH assay (see Table 1). Moreover, combining iPTH with other biomarkers did not improve the diagnostic accuracy. It is of clinical relevance to note that the association between the bone phenotype and iPTH appears to be different for Afro-American dialysis patients [30]. For the same value of iPTH, low turnover bone disease is more prevalent in Afro-Americans [31].

**Table 1:** Area under receiver operating curves of circulating bone biomarkers to distinguish high and low bone turnover from nonhigh and nonlow bone turnover, respectively, as assessed by BFR/BS [27] (approval pending)

Blood sample marker	N	AUROC (95% CI)	Best cut off
Low vs non low			
iPTH (pg/ml)	280 vs 196	0.701 (0.653–0.750)	103.8
wPTH (pg/ml)	260 vs 180	0.712 (0.662–0.761)	48.0
bALP (U/l)	273 vs 190	0.757 (0.713–0.801)	33.1
P1NP (ng/ml)	280 vs 1197	0.650 (0.599–0.701)	498.9
Combined iPTH + bALP	272 vs 188	0.718 (0.670–0.767)	NA
Combined wPTH + bALP	257 vs 174	0.743 (0.695–0.790)	NA
High vs non high			
iPTH (pg/ml)	81 vs 395	0.724 (0.663–0.786)	323.0
wPTH (pg/ml)	75 vs 365	0.678 (0.611–0.746)	61.4
bALP (U/l)	77 vs 386	0.711 (0.655–0.767)	42.1
P1NP (ng/ml)	81 vs 396	0.743 (0.689–0.797)	621.1
Combined iPTH + bALP	76 vs 384	0.718 (0.658–0.779)	NA
Combined wPTH + bALP	72 vs 359	0.691 (0.628–0.725)	NA

*AUROC* area under the receiver operating characteristic curve, *bALP* bone-specific alkaline phosphatase, *BFR/BS* bone formation rate/bone surface, *CI* confidence in-terval, *iPTH* intact parathyroid hormone, *NA* not available, *P1NP* amino-terminal propeptide of type 1 procollagen, *wPTH* whole parathyroid hormona

**Fig. 3:**
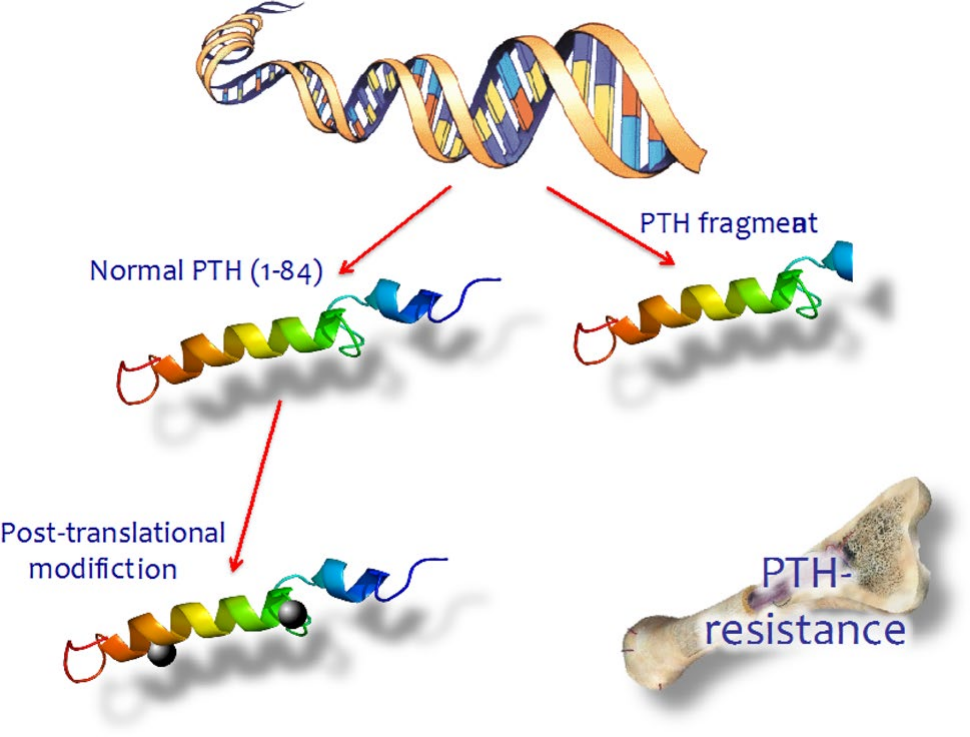
Components of PTH resistance in CKD. Secreted PTH from parathyroid cells may exist as a bio-active 1–84 fragment containing polypeptides, but also as variable amounts of PTH fragments with variable biological effects, including an antagonizing impact. In addition, after secreting normal PTH, in CKD the hormone may undergo abnormal posttranslational modification. Finally, target tissue may be hypo-responsive to normal PTH in CKD

The explanation for the moderate performance of PTH as an indicator of bone turnover is several fold. First, as mentioned above, in CKD the serum concentration of PTH generally overestimates its biological activity due to posttranslational modification, especially oxidation, of the hormone, which renders it biologically inert [10]. In addition, the existence of PTH hypo-responsiveness at the target tissue level also limits the association between PTH concentration and its bioactivity. Accumulation of inhibiting C-terminal fragments, too, may contribute to this partial PTH resistance [32] (Fig. 3). A frequently neglected issue is the fact that there is no evidence indicating a feedback control between bone turnover and PTH secretion. The spectrum of adynamic bone disease in uremia is much wider than just low concentration of PTH due to iatrogenic oversuppression of the hormone. The uremic retention molecule indoxyl sulphate, for instance, promotes osteoblast apoptosis [33], FGF23 inhibits normal Wnt signaling pathways by osteoblasts [34], while increased concentrations of asymmetric dimethyl arginine (ADMA), an inhibitor of nitric oxide generation, and elevated in CKD, hampers osteoblastic differentiation [35], as has been described for acidosis [36]. Moreover, chronic inflammation may contribute to abnormal bone turnover, as is well established in rheumatologic diseases [37]. While all these factors may contribute to adynamic bone disease in CKD, they will hardly suppress PTH secretion. This, therefore, may in part explain the ever-increasing prevalence of adynamic bone disease, especially in non-black patients on dialysis [38], but also the limited ability of PTH to predict low turnover bone disease.

With all these pitfalls in mind, it is important to stress that one of the largest and most recent analyses confirmed that PTH currently is the most useful biomarker for bone turnover in CKD [27].

## Bone-specific alkaline phosphatase

Slightly less than 50% of circulating alkaline phosphatase is bone-derived (BSAP), and the remainder mainly originates from hepatocytes [39]. In the absence of cholestatic liver disease, arbitrarily defined as normal concentration of γGT, the value of total alkaline phosphatase above the normal range, can arguably be considered as reflecting BSAP [25]. BSAP is produced by osteoblasts during bone formation and an important role is to inactivate pyrophosphate, an inhibitor of mineralization [40]. Recent small studies indicate a potential role for BSAP isotypes where, for instance, the B1x subtype was positively associated with low bone turnover disease, so improving diagnostic accuracy for that diagnosis [41]. BSAP is considered to reflect bone turnover, in particular the bone formation rate. Both BSAP and total AP are associated with all-cause and cardiovascular mortality in dialysis patients [42, 43], but also with fracture risk [43]. This association of BSAP with fracture risk, however, was not found in a non-CKD population [44, 45]. In a study of 42 hemodialysis patients, BSAP showed a better correlation with bone turnover (histomorphometrically determined) than total AP, and outperformed iPTH in detecting high bone turnover [46]. However, the aforementioned study by Sprague et al., combining databases from four countries, found that BSAP (cut-off value 33.1 U/l) was only slightly better than PTH for diagnosing low turnover disease, but not for high turnover disease (Table 1) [27]. Importantly, this latter study did not support the combined use of BSAP and PTH, as shown in Table 1. Clinically, however, these data on BSAP can still be very useful. The PPV for low bone turnover disease, for instance, can easily be increased by applying a considerably lower cut-off value for BSAP, and vice versa for high-turnover disease by considering a value above 42.1 U/l (Table 1).

## N-terminal propeptide of procollagen-1

The protein matrix of bone consists to a large extent of collagen 1. Collagen 1 is formed by osteoblasts as procollagen-1, which forms a triple helix (combining two α- and one β-chain) on organization into its quaternary structure. On maturation both the N-terminal and C-terminal endings are cleaved, leaving the triple helix collagen 1, a longitudinal matrix component along which mineralization is organized [47]. The small cleavage fragments, N-terminal propeptide of procollagen type 1 (P1NP) and C-terminal propeptide of procollagen 1 (P1CP) are detectable in the circulation and are therefore indicative of the formation rate of bone collagen [48]. Since these cleavage products originate from a triple helix, they initially are trimeric compounds that are rapidly broken down to monomeric peptides. The latter is of special importance in CKD, because the monomeric form of P1NP accumulates in kidney disease, while the trimeric form does not [49]. When using P1NP, it is therefore important to know the specific assay characteristics, because the intact P1NP assay is the only reliable one in CKD. P1CP, in particular, has a short half-life, hence P1NP is recommended as the bone formation marker in the general population [48].

P1NP was also assessed in the study by Sprague et al. cited above in which biomarkers were validated based on bone histomorphometry [27]. As shown in Table 1, in that study, P1NP performed worse than iPTH or BSAP in distinguishing between low versus non-low bone turnover, and had no additional value over iPTH for diagnosing high turnover bone disease. The assay that was used was one that detects total P1NP (personal communication from lead author, S. Sprague) leaving the possibility that the intact P1NP assay performs better in CKD populations.

## C-terminal crosslaps of collagen 1

Mature triple helices formed from collagen 1 are crosslinks by non-collagenous proteins (pyridinolines and deoxypyridinolines), also formed by osteoblasts, to establish a firm longitudinally oriented protein network. During bone degradation, lysosomal enzymes derived from osteoclasts including tartrate-resistant acid phosphatases (TRAP) and cathepsin K are responsible for breakdown of the collagenous matrix of bone at specific sites, yielding both carboxy- and nitrogen telopeptide containing parts of the original collagen 1 (CTX and NTX) but also the cross-linking protein [48]. The assay used for CTX determines the specific amino acid sequence of the telopeptide of collagen 1 (this telopeptide is termed the crosslap, in the case of β isomerization of aspartic acid: β crosslaps). Importantly, as bone ages, α-aspartic amino acid converts to β-aspartic acid, and therefore the detecting of βCTX is indicative of resorption of matured bone [50]. Unfortunately, CTX has kinetic characteristics that significantly limit its clinical usefulness in CKD. First, there is a relevant circadian rhythm, but importantly its removal from the circulation is highly dependent on kidney function [48]. For this reason, the use of CTX cannot be recommended in patients with CKD.

## Tartrate-resistant acid phosphate 5b

TRAP5b is a mainly osteoclast-derived enzyme [51, 52]. As its name indicates, this protein cleaves phosphate from proteins thereby influencing their function. Since its activity is optimal at a relatively low pH, it is active at acidic sites such as in resorption lacunae in bone. Both osteopontin and bone sialoproteins are presumed targets of the enzyme [51], but also collagen 1 itself [48]. Cell lines, differentiated in vitro by osteoclastic-like cells by exposure to receptor activator of nuclear factor κ-B ligand (RANKL, in physiology an osteoblast-derived ligand) produce TRAP5b and its amount is strongly associated with both the number and size of the osteoclast-like cells [53]. Interestingly TRAP5b is not affected by CKD [54], nor even by hemodialysis or peritoneal dialysis [55, 56]. Based on these characteristics, TRAP5b is a most attractive candidate biomarker for bone resorption in patients with CKD, unlike βCTX. The compound can be measured in serum by immunoassays [52]. However, currently data are lacking that indicate its association with bone histomorphometry.

## Conclusion

Biomarkers of bone turnover are promising aids in clinical nephrology practice. A thorough knowledge of what they indicate, of the assay characteristics and of the impact of low estimated glomerular filtration rate (eGFR) are important in interpreting the results. Generally, biomarkers lack sufficient specificity to be able to base far-reaching treatment decisions upon them. However, as follow-up parameters, they may be very useful. Most biomarkers provide mainly an indication of bone formation and, as such, PTH, BSAP and also P1NP can be used. The only useful biomarker that reflects bone resorption in patients with CKD is TRAP5b, but the concentration of this enzyme has not yet been validated with respect to the gold standard, which is bone histomorphometry.
